# Secondary Rashes in Scarlet Fever

**Published:** 1897-03

**Authors:** J. O. Symes


### SECONDARY RASHES IN SCARLET FEVER.

BY

J. O. Symes, M.D. Lond.

---

Secondary rashes during the convalescent stage of scarlet fever are by no means of uncommon occurrence. They are not to be confounded with the true relapse in which the patient passes again through the stages of vomiting, sore-throat, strawberry tongue, rash, and desquamation. Such relapses are said to occur as frequently as once in every two hundred cases, are most common during the second or third week of the illness, and are seldom severe in character. The eczema consequent upon irritating discharges from the nose or ears, and which frequently in children results in impetigo, is as a rule, unless indeed it spread to the head, easily controlled.

Dr. F. F. Caiger[1] found eczema to be present in 3.3 per cent. of 1,008 cases. The true secondary rashes, however, are of more importance, as they frequently indicate the commencement of a period fraught with grave danger to the patient. Cases of exceptional severity were reported by Mr. Manning in his article in the *Lancet*, August 13th, 1892, entitled, "The Skin Eruptions which occur in Septicæmia following Scarlet Fever and Diphtheria." This complication was present in 2.1 per cent. of 6,000 cases, and in 4.8 per cent. of the children under five years of age. The rash was at various times papular, blotchy, or macular, and almost invariably associated with sloughing of the fauces, wasting, and a rise in the temperature. The attacks which commenced during the second and third weeks of convalescence were in many cases fatal. As is indicated in the title of Mr. Manning's paper, there would seem here to be an element of septic poisoning derived probably from the throat, and in this point the cases differ from those recorded below, with which otherwise they have much in common. These came under my observation while I was in residence at the London Fever Hospital. Secondary rashes may be erythematous, urticarial, eczematous, papular, or hemorrhagic. The following cases are illustrative of these varieties :—

Case 1.—Commenced with vivid rash, much membrane on fauces, and delirium. On the 18th day there was an erythematous rash covering trunk and arms. (20th day) Rigor, vomiting, adenitis, trace of albumen in urine. The temperature fell after seven days, and recovery was satisfactory.

[illegible]ase 2.—On 10th day of illness had slight return of tonsillitis, wh[illegible] subsided. (26th day) Erythema on chest, vomiting, tonsillitis, alb[illegible]nuria, fever. The rash was visible for forty-eight hours, and the [illegible]perature remained up for four days. Recovery complete.

Case 3.—Had severe tonsillitis. On 15th day erythema covered chest and abdomen. (18th day) Urticaria, chiefly on arms and legs. Some painful enlargement of cervical lymphatic glands. No rise of temperature.

Case 4.—(17th day) Adenitis, with rise of temperature. (20th day) Urticaria. The rash was troublesome for twenty-four hours, but never returned.

Case 5.—Initial throat affection severe. (12th day) Trace of albumen in urine. (14th day) Tonsillitis. (16th day) Eczema over bony prominences of knees, elbows, ankles, and scapulæ. No rise of temperature. Convalescence delayed by continued slight albuminuria.

[1] *Lancet*, 1891, i. 1249.

Case 6.—On 18th day there was eczema on legs and inner side of knees, and during three days previously a trace of albumen in urine. (23rd day) Tonsillitis, nephritis, fever. (47th day) Erythema on trunk and arms. Convalescence delayed until 73rd day.

Case 7.—The initial rash was finely vesicular, and irritating. The fauces were very little congested, and showed no exudation. (14th day) Slight puffiness over cervical lymphatic glands was noticed. (18th day) Dull red papules on extensor surfaces of arms and legs, most marked at flexures of joints, and spreading between the fingers. On pressure, faint punctate hemorrhagic spots were left. There was no irritation of skin. (20th day) Headache and albuminuria. The rash did not disappear until the 32nd day, and in the interval had become blotchy. Slight rise of temperature.

Case 8.—The commencement was marked by severe tonsillitis and rheumatism. (24th day) There appeared a papular rash on extensor surfaces of arms, legs, and thighs, and upon the back. It extended on the following day between the fingers and to the soles of the feet, and gave rise to much itching and discomfort. The appearance of the rash was accompanied by a slight rise of temperature, and there was a faint cloud of albumen in the urine from the 14th day.

Case 9.—Rheumatism and much exudation on the tonsils during early stages. Trace of albumen on 6th and subsequent days. (17th day) Headache, pyrexia, nephritis. (20th day) A red, hard, papular rash on extensor surfaces of arms and legs, and on chest. Much itching. This extended to the hands and feet, but by the 23rd day had disappeared. (60th day) The rash returned, the quantity of blood and albumen in the urine increased, and adenitis commenced. Convalescence was completed on the 89th day.

Case 10.—Much exudation on fauces, accompanied by adenitis. On 52nd day, when recovery was apparently completed, an erythematous rash appeared on chest, some raised red papules and minute purpuric spots upon the limbs. There was no adenitis, albuminuria, or rise of temperature. On 59th day there was severe vomiting, but no further complications arose.

Most of the rashes appeared in or about the third week of the illness, and almost invariably in those patients in whom the initial throat symptoms had been of more than average severity. Although the appearance of the skin-lesions frequently coincided with a recrudescence of tonsillitis, it does not seem likely that they were the result of septic absorption from the fauces. In some of the cases no tonsillitis was observed; in none of them was there ulceration or sloughing; whilst in others the rash preceded the appearance of tonsillar trouble. The more probable explanation would appear to be that there is about the third week a critical period during which certain products of the disease are being eliminated, and that in this process the organs engaged are liable to suffer. In the digestive system such disturbance is marked by diarrhœa and vomiting, and in the kidneys by nephritis and albuminuria, the latter of so slight a nature, perhaps, as to be disregarded. The lymphoid tissue of tonsils and the lymphatic glands are noticeably excitable, and as we should expect, that great excretory organ, the skin, is frequently affected. The septic rashes described by Manning were accompanied by much wasting, and the mortality was very high. None of the cases described in this paper ended fatally, nor was there wasting or other symptom usually associated with septicæmia. Mahomed observed that in scarlet fever there were certain changes which were of constant occurrence between the 18th and 22nd day of the illness. Such were, a rise of arterial tension, a diminished excretion of urine and urea, a rise of temperature, albuminuria, otorrhœa, adenitis, and diarrhœa. He makes no mention of secondary rashes, and indeed the subject has received but little attention. There can, however, be no doubt that their presence is far from uncommon; they would be still more frequently observed were they diligently enquired for. The rash is so frequently the precursor of grave complications, that great importance should be attached to its detection. By so doing we are enabled to change the diet and habit of life of the patient, and by a free use of such drugs as calomel and acid tartrate of potassium we may hope, as Mahomed has shown, to ward off or minimise a threatening attack of nephritis, or other complication.

Erythematous, urticarial, and purpuric rashes are frequently seen in scarlet fever complicated by acute rheumatism; but in the above series of cases rheumatism was only noted in Nos. 8 and 9. The eruption in the last four cases reported closely resembled "urticaria papulosa." Small crops of papules showed a tendency to run together, and on pressure the colour faded from the circumference of the papule, showing a minute red spot in the middle.

In each case the management had been similar, the patient being allowed to rise on the 10th day, and put on a meat diet at the end of a fortnight. From many reasons it would not seem likely that such management was in any way responsible for the subsequent complications. Chill is no longer commonly regarded as a serious factor in the causation of nephritis.

Patients frequently become dropsical and albuminuric whilst still in bed, and there is no reason why chill should act more potently at one time (*e.g.* 18th—21st day) than at another. Mahomed's observations were made on patients who were kept in bed and on a milk diet for twenty-one days. Caiger, however, found that eczema was more common amongst the patients on a full meat diet. These facts would point to the conclusion that the occurrence of complications is uninfluenced by the length of stay in bed or by the quality of the diet. There is during the latter half of the third week a critical period during which certain products of the disease are eliminated, and during the process of excretion these bodies may excite various pathological conditions in lymphatic tissues, kidneys, and skin. The appearance of various rashes, especially that resembling "urticaria papulosa," is often the first indication of the onset of more serious complications, and thus affords us an early opportunity of commencing treatment with a view to their prevention or mitigation.

